# Association between dementia and discharge status in patients hospitalized with pneumonia

**DOI:** 10.1186/s12890-017-0473-8

**Published:** 2017-10-06

**Authors:** Taisuke Jo, Hideo Yasunaga, Yusuke Sasabuchi, Nobuaki Michihata, Kojiro Morita, Yasuhiro Yamauchi, Wakae Hasegawa, Hideyuki Takeshima, Yukiyo Sakamoto, Hiroki Matsui, Kiyohide Fushimi, Takahide Nagase

**Affiliations:** 1 Department of Health Services Research, Graduate School of Medicine, The University of Tokyo, 7-3-1 Hongo, Bunkyo-ku, Tokyo, 1130033 Japan; 2 Department of Respiratory Medicine, Graduate School of Medicine, Tokyo, Japan; 3 Department of Clinical Epidemiology and Health Economics, School of Public Health, The University of Tokyo, Tokyo, Japan; 4 Department of Health Policy and Informatics, Tokyo Medical and Dental University Graduate School of Medicine, Tokyo, Japan

**Keywords:** Cognition, Cohort studies, Hospital mortality, Patient discharge, Respiratory tract infection

## Abstract

**Background:**

Pneumonia is the most common cause of death in patients with dementia, but the outcomes of patients with dementia hospitalized with pneumonia are poorly understood. We sought to illuminate the association between dementia and in-hospital mortality and discharge status in patients hospitalized with pneumonia.

**Methods:**

We used the Diagnosis Procedure Combination database, a national inpatient database in Japan, to identify retrospectively patients aged ≥60 years admitted to hospital with pneumonia during the study period of May 1, 2010 to March 31, 2014. We recorded their sex, age, body mass index, severity of pneumonia and comorbidities (including dementia). The outcomes were in-hospital mortality and discharge home. Multivariable Cox regression analysis was performed to analyze factors influencing discharge home.

**Results:**

We identified 470,829 patients hospitalized with pneumonia; 45,031 were recorded as having dementia (9.6%). In-hospital mortality was 13.1% and 13.4% in patients with and without dementia, respectively (*P* = 0.63). The proportions of patients discharged home were 52.9% and 71.3% in patients with and without dementia, respectively (*P* < 0.001). The adjusted hazard ratio for discharge home for patients with dementia was 0.68 (95% confidence interval, 0.67–0.69; *P* < 0.001).

**Conclusions:**

In-hospital mortality from pneumonia did not differ significantly between patients with and without dementia; however, those with dementia were less likely to be discharged home.

## Background

Pneumonia and dementia are major concerns as societies age worldwide [1, 2]. They frequently coexist [3]; pneumonia is the most common cause of death in patients with dementia [4, 5], and dementia is a well-recognized risk factor for community acquired pneumonia [6] and aspiration pneumonia [7] in the elderly. Pneumonia is also reportedly associated with poor long-term prognosis in patients with dementia [8, 9]. Several studies have assessed the association between independent comorbidities with in-hospital mortality [10–12] in patients with pneumonia; however, the influence of dementia is not completely understood.

Patients with dementia frequently have impaired ability to engage with activities of daily living, and may be bedridden before or after the diagnosis of pneumonia, potentially resulting in failure to be discharged home after hospitalization. Factors associated with discharge home has been intensively studied in stroke patients [13–15]. Dysphagia and cognitive deficit have been reported as risk factors for discharge disposition [13, 14]. Whereas, to the best of our knowledge, the relationship between dementia and discharge status in elderly patients with pneumonia has not been examined.

We used a national inpatient database in Japan to establish whether a diagnosis of dementia influences in-hospital prognosis or discharge status in elderly patients with pneumonia.

## Methods

### Data source and patient selection

Ours was a retrospective cohort study using the Japanese Diagnosis Procedure Combination (DPC) database [16, 17], which comprises administrative claims data and discharge abstract data. It contains main diagnosis, primary diagnosis on admission, comorbidities present on admission and comorbidities diagnosed during each episode of hospitalization, recorded using International Classification of Diseases and Related Health Problems, 10th Revision (ICD-10) codes with corresponding text data in Japanese. Comorbidities are scored according to the updated Charlson comorbidity index (CCI) [18]. The database also records each patient’s age, sex, height and weight, prescription records, intensive care unit admission and discharge status, and the following information required to quantify the severity of pneumonia in accordance with the A-DROP system [19, 20]: blood urea nitrogen (BUN), peripheral oxygen saturation (SpO_2_) while breathing a fraction of inspired oxygen <0.35 or ≥0.35, level of consciousness and systolic blood pressure. Level of consciousness was assessed using the Japan Coma Scale. Japan Coma Scale score 0 denoted alert consciousness; scores 1–3 denote delirium; scores 10–30 denote somnolence; and scores 100–300 denote coma [21]. In the present study, Japan Coma Scale scores ≥1 were defined as “impaired consciousness”.

We identified patients aged ≥60 years hospitalized with pneumonia (ICD-10 code J10 − J18 or J69) between May 1, 2010 and March 31, 2014. Among these patients, those with dementia were identified by the ICD codes for ‘dementia’ (F00–F03 or G30) or the prescription of donepezil, galantamine, rivastigmine or memantine. Patients with cancer and dysphagia was identified by ICD codes for ‘Neoplasm’ (C) and ‘dysphagia’ (R13), respectively.

### Ethical considerations

Conduct of the study was approved by the Institutional Review Board of The University of Tokyo, which waived the requirement for informed consent owing to the anonymity of the data.

### Outcome

The study outcome was discharge status (discharge home, discharge to another facility or all-cause in-hospital death).

### Statistical analysis

Body mass index (BMI) was grouped into the following six categories according to the World Health Organization body mass index (BMI) classification: <15.9, 16.0–16.9, 17.0–18.4, 18.5–22.9, 23.0–24.9, 25–29.9, 30.0–34.9 and ≥35.0 kg/m^2^ [22]. Patients with missing data for height and/or weight were categorized as missing. Pearson’s chi squared test was used to compare proportions of categorical variables between patients with and without dementia hospitalized with pneumonia. Kaplan-Meier analysis followed by Cox proportional hazards regression analysis using length of hospitalization as time variable was performed and the variables influencing discharge home were assessed. Discharge to other facilities and in-hospital death were regarded as censored. The independent variables included dementia, age, sex, BMI, total A-DROP score or ADROP items (BUN >21 mg/dl, SpO_2_ < 90%, impaired consciousness, systolic blood pressure < 90 mmHg), cancer, hemodialysis and intensive care unit admission. We classified A-DROP score as follows; mild: 0, moderate: 1-2, severe: 3, most severe: 4-5 or systolic blood pressure < 90 mmHg. Multiple imputation method was utilized to impute missing values in BMI and A-DROP items [23, 24]. Multivariate imputation by chained equations technique was applied to obtain 20 imputed datasets by using the following covariates: dementia, age, sex, CCI, cancer, hemodialysis and intensive care unit admission. Estimates from the 20 imputed datasets where then combined by fitting into Rubin’s rule for acquisition of combined imputation estimates and standard errors [25]. Statistical analyses were performed using SPSS version 22.0 (IBM Corp., Armonk, NY, USA) and Stata/MP version 14 (StataCorp, College Station, TX, USA).

## Results

We identified a total of 470,829 patients aged ≥60 years hospitalized with pneumonia during the study period: of these, 45,031 (9.6%) had dementia and 100,198 (21.3%) were admitted more than once. Dysphagia and nasogastric tube feeding was observed in 4953 (11.0%) and 4120 (9.1%) patients with dementia, respectively, and 19,980 (4.7%) and 22,123 (5.2%) patients without dementia, respectively. Of those admitted more than once, 9118 (9.1%) had dementia and 91,080 (90.9%) did not.

Table 1 shows the demographic and clinical characteristics of patients hospitalized with pneumonia. Age, sex, BMI, A-DROP score, CCI and cancer differed significantly between patients with and without dementia. In particular, patients with dementia were younger and had lower BMI compared to those without dementia. Missing values of BMI were observed in 22.0% and 16.1% of Patients with and without dementia, respectively. Values of BUN, SpO_2_, consciousness and systolic blood pressure were missing in 1.8% and 2.2%, 1.9% and 2.3%, 3.2% and 2.3% and 1.6% and 2.1% in patients with and without dementia, respectively.

**Table 1:** Characteristics of hospitalized pneumonia patients with and without dementia

	Total (*n* = 412,844)	Dementia (*n* = 40,144)	Non-dementia (*n* = 372,700)	*P*
Age (years), %				<0.001
60–69	14.9	2.2	16.3	
70–79	29.0	17.0	29.0	
80–89	40.4	55.4	40.4	
≥ 90	15.6	25.3	15.6	
Sex (female), %	41.9	54.5	40.6	<0.001
Body mass index (kg/m^2^),%				<0.001
< 15.9	9.1	11.5	8.9	
16.0–16.9	5.6	6.6	5.5	
17.0–18.4	10.9	12.2	10.8	
18.5–22.9	35.8	33.3	36.0	
23.0–24.9	10.8	7.7	11.2	
25–29.9	9.5	5.9	9.8	
30.0–34.9	1.4	0.7	1.4	
≥ 35.0	0.3	0.1	0.3	
Missing	16.6	22.0	16.1	
A-DROP, %				<0.001
Mild	9.6	1.3	10.4	
moderate	61.7	63.0	61.5	
severe	15.6	20.1	15.1	
most severe	13.2	15.7	13.0	
Charlson Comorbidity Index, %				<0.001
0	41.3	10.2	44.6	
1-–2	43.5	55.9	42.2	
≥ 3	15.2	34.0	13.2	
Cancer, %	12.4	7.0	13.0	<0.001
Hemodialysis, %	1.7	1.1	1.7	<0.001
Intensive care unit admission, %	3.3	3.4	3.3	0.783

Discharge status is presented in Table 2. The proportion of patients with dementia discharged home was significantly lower than the proportion of non-dementia patients (52.9% versus 71.3%, respectively; *P* < 0.001). There was no significant difference in all-cause in-hospital mortality between the groups (13.1% in those with dementia versus 13.4% in those without, *P* = 0.65).

**Table 2:** Discharge status after hospitalization with pneumonia

	Total (*n* = 412,884)	Dementia (*n* = 40,144)	Non-dementia (*n* = 372,700)	*P*
Discharge status, n (%)				<0.001
Discharge to home	287,028 (69.5)	21,249 (52.9)	265,779 (71.3)	
Discharge to other facility	66,556 (16.1)	12,841 (32.0)	53,715 (14.4)	
In-hospital death	55,333 (13.4)	5260 (13.1)	50,073 (13.4)	
Not specified	3927 (1.0)	794 (2.0)	3133 (0.8)	

**Fig. 1:**
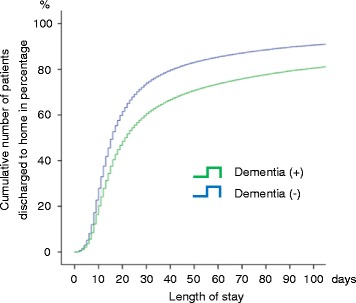
Patients discharged home after hospitalization with pneumonia, according to length of hospital stay. Cumulative number of patients discharged to home shown in percentage for patients hospitalized with pneumonia with dementia (green curve) and without dementia (blue curve). Regardless of the length of hospitalization, a smaller proportion of patients with dementia were discharged home

In the Cox proportional hazards regression model, the adjusted hazard ratio for discharge home was 0.68 (95% confidence interval 0.67–0.69, *P* < 0.001) for those with dementia with reference to those without. Age, A-DROP items (BUN, SpO_2_, level of consciousness, systolic blood pressure), cancer, hemodialysis and intensive care unit admission was significantly associated with reduction in discharge home, while higher BMI was significantly associated with increase in discharge home (Table 3). The proportion of patients with dementia discharged home after hospitalization with pneumonia was significantly lower, regardless of the length of hospitalization (Fig. 1).

**Table 3:** Multivariable Cox regression analysis for discharge home

	Hazard ratio	95% confidence interval	*P*
Dementia	0.68	0.67–0.69	<0.001
Age (years)			
60–69	Reference		
70–79	0.82	0.81–0.83	<0.001
80–89	0.66	0.65–0.67	<0.001
≥ 90	0.51	0.51–0.52	<0.001
Sex (female)	1.005	1.00–1.01	0.16
Body mass index (kg/m^2^)			
< 18.5	0.75	0.74–0.75	<0.001
18.5–22.9	Reference		
23.0–24.9	1.13	1.11–1.14	<0.001
25–29.9	1.16	1.15–1.18	<0.001
≥ 30.0	1.10	1.07–1.14	<0.001
BUN >21 mg/dl	0.79	0.78–0.79	<0.001
SpO2 < 90%	0.72	0.71–0.72	<0.001
Impaired consciousness	0.59	0.58–0.59	<0.001
Systolic blood pressure < 90 mmHg	0.71	0.69–0.72	<0.001
Cancer	0.83	0.82–0.84	<0.001
Hemodialysis	0.87	0.85–0.90	<0.001
Intensive care unit admission	0.49	0.48–0.50	<0.001

Abbreviations: *BUN* blood urea nitrogen; *SpO*_*2*_ peripheral oxygen saturation; *FiO*_*2*_ fraction of inspired oxygen. Multiple imputation was used for missing values for body mass index (BMI), BUN >21 mg/dl, SpO2 < 90%, Impaired consciousness, Systolic blood pressure < 90 mmHg

## Discussion

We found that all-cause in-hospital mortality after hospitalization for pneumonia was not significantly different between elderly patients with or without dementia, but those with dementia were significantly less likely to return home.

Bronchopneumonia is reportedly the most common cause of death in patients with dementia disorders [4]. Previous reports on dementia and in-hospital mortality in elderly people with pneumonia showed mixed results. Several studied showed patients with dementia were at higher risk of mortality after pneumonia [9, 26], whereas a previous prospective study showed impaired cognitive function was not significantly associated with in-hospital death in patients aged ≥75 years [12]. In our study, patients with dementia hospitalized with pneumonia were not necessarily at a higher risk of death.

As with the rest of the world, the importance of discharge home is increasingly recognized in Japan, allowing patients to benefit from home care in familiar surroundings. Home care is an increasingly important part of continuous care for seniors. Home care may reduce the risk of infection with antimicrobial-resistant micro-organisms [27] and delirium [28] compared with residential and nursing facilities. Furthermore, previous studies have shown an association between placement other than at home after hospitalization and 2-year mortality in the elderly [29]. There was another study showing the advantage of home and community-based care over nursing homes, in survival [30]. Besides, home and community-based services are well recognized to reduce long-term care spending in the United States [31, 32].

A diagnosis of dementia appears to condemn hospitalized patients to be discharged to another medical facility rather than home [33, 34]. In a previous large cross-sectional survey in Japan, patients with dementia were more likely to be discharged from an intermediate care facility to places other than home [35]. Another study from the United States also showed the influence of dementia on reduced likelihood of returning home after hospitalization [36]. Our study showed that patients with dementia were significantly less likely to be discharged home after hospitalization with pneumonia, regardless of length of stay. The reason for this finding remains unclear. Lung disease and impaired lung function are reportedly associated with inferior cognitive performance [37–40]. We speculate that impaired lung function brought about by pneumonia in patients with dementia may lead to a further decline in cognitive function, making it less likely that a patient will be able to return home. In the present study, dementia patients were more likely to have dysphagia. Reportedly, dysphagia was associated with increase in aspiration pneumonia [41] and discharge disposition [13, 14] in stroke patients. Thus, the association between dementia and discharge home in the present study can be partly explained by the high proportion of dysphagia in the dementia patients.

Our study had some limitations. First, the diagnosis of dementia was not necessarily certified by clinicians, so may have been prone to inaccuracy. Secondly, the etiology and severity of dementia, and the etiology of pneumonia, were not taken into consideration. Thirdly, family environment and support from the community were unable to be assessed.

## Conclusions

In conclusion, all-cause in-hospital mortality of patients hospitalized with pneumonia did not appear to be significantly influenced by dementia. However, patients with dementia hospitalized with pneumonia were less likely to return home than those without dementia, even after adjustment for other factors such as age, BMI and severity of pneumonia.

## Abbreviations

BMI: Body mass index
BUN: Blood urea nitrogen
CCI: Charlson comorbidity index
DPC: Diagnosis Procedure Combination
FiO_2_: Fraction of inspired oxygen
ICD-10: International Classification of Diseases and Related Health Problems, 10th Revision
SpO_2_: Level of oxygen saturation

## References

[1] Thannickal VJ, Murthy M, Balch WE, Chandel NS, Meiners S, Eickelberg O, Selman M, Pardo A, White ES, Levy BD, Busse PJ, Tuder RM, Antony VB, Sznajder JI, Budinger GR (2015). Blue journal conference. Aging and susceptibility to lung disease. Am J Respir Crit Care Med.

[2] Savva GM, Arthur A (2015). Who has undiagnosed dementia? A cross-sectional analysis of participants of the Aging, Demographics and Memory Study. Age Ageing.

[3] Bauer K, Schwarzkopf L, Graessel E, Holle R (2014). A claims data-based comparison of comorbidity in individuals with and without dementia. BMC Geriatr.

[4] Brunnstrom HR, Englund EM (2009). Cause of death in patients with dementia disorders. Eur J Neurol.

[5] Magaki S, Yong WH, Khanlou N, Tung S, Vinters HV (2014). Comorbidity in dementia: update of an ongoing autopsy study. J Am Geriatr Soc.

[6] Torres A, Peetermans WE, Viegi G, Blasi F (2013). Risk factors for community-acquired pneumonia in adults in Europe: a literature review. Thorax.

[7] Manabe T, Teramoto S, Tamiya N, Okochi J, Hizawa N (2015). Risk factors for aspiration pneumonia in older adults. PLoS One.

[8] Salive ME, Satterfield S, Ostfeld AM, Wallace RB, Havlik RJ (1993). Disability and cognitive impairment are risk factors for pneumonia-related mortality in older adults. Public Health Rep.

[9] Foley NC, Affoo RH, Martin RE (2015). A systematic review and meta-analysis examining pneumonia-associated mortality in dementia. Dement Geriatr Cogn Disord.

[10] Cascini S, Agabiti N, Incalzi RA, Pinnarelli L, Mayer F, Arca M, Fusco D, Davoli M (2013). Pneumonia burden in elderly patients: a classification algorithm using administrative data. BMC Infect Dis.

[11] Rothberg MB, Pekow PS, Priya A, Zilberberg MD, Belforti R, Skiest D, Lagu T, Higgins TL, Lindenauer PK (2014). Using highly detailed administrative data to predict pneumonia mortality. PLoS One.

[12] Calle A, Marquez MA, Arellano M, Perez LM, Pi-Figueras M, Miralles R (2014). Geriatric assessment and prognostic factors of mortality in very elderly patients with community-acquired pneumonia. Arch Bronconeumol.

[13] Arnold M, Liesirova K, Broeg-Morvay A, Meisterernst J, Schlager M, Mono ML, El-Koussy M, Kägi G, Jung S, Sarikaya H. Dysphagia in Acute Stroke: Incidence, Burden and Impact on Clinical Outcome. PLoS One. 2016;11:e0148424. 10.1371/journal.pone.0148424 PMC4749248 26863627

[14] Nguyen VQ, PrvuBettger J, Guerrier T, Hirsch MA, Thomas JG, Pugh TM, Rhoads CF 3rd. Factors associated with discharge to home versus discharge to institutional care after inpatient stroke rehabilitation. Arch Phys Med Rehabil. 2015;96:1297–303. 10.1016/j.apmr.2015.03.007 25823940

[15] Ifejika-Jones NL, arun N, Peng H, Elizabeth A, Grotta JC, Francisco GE (2012). The interaction of aspiration pneumonia with demographic and cerebrovascular disease risk factors is predictive of discharge level of care in acute stroke patient. Am J Phys Med Rehabil.

[16] Yasunaga H, Matsui H, Horiguchi H, Fushimi K, Matsuda S (2013). Clinical epidemiology and health services research using the Diagnosis Procedure Combination Database in Japan. Asian Pac J Dis Manage.

[17] Hasegawa W, Yamauchi Y, Yasunaga H, Sunohara M, Jo T, Matsui H, Fushimi K, Takami K, Nagase T (2014). Factors affecting mortality following emergency admission for chronic obstructive pulmonary disease. BMC Pulm Med..

[18] Quan H, Li B, Couris CM, Fushimi K, Graham P, Hider P, Januel JM, Sundararajan V (2011). Updating and validating the Charlson comorbidity index and score for risk adjustment in hospital discharge abstracts using data from 6 countries. Am J Epidemiol.

[19] Miyashita N, Matsushima T, Oka M, Japanese Respiratory Society (2006). The JRS guidelines for the management of community-acquired pneumonia in adults: an update and new recommendations. Intern Med.

[20] Yamauchi Y, Yasunaga H, Matsui H, Hasegawa W, Jo T, Takami K, Fushimi K, Nagase T (2015). Comparison of clinical characteristics and outcomes between aspiration pneumonia and community-acquired pneumonia in patients with chronic obstructive pulmonary disease. BMC Pulm Med.

[21] Shigemori M, Abe T, Aruga T, Ogawa T, Okudera H, Ono J, Onuma T, Katayama Y, Kawai N, Kawamata T, Kohmura E, Sakaki T, Sakamoto T, Sasaki T, Sato A, Shiogai T, Shima K, Sugiura K, Takasato Y, Tokutomi T, Tomita H, Toyoda I, Nagao S, Nakamura H, Park YS, Matsumae M, Miki T, Miyake Y, Murai H, Murakami S, Yamaura A, Yamaki T, Yamada K, Yoshimine T (2012). Guidelines for the management of severe head injury, 2nd edition guidelines from the Guidelines Committee on the Management of Severe Head Injury, the Japan Society of Neurotraumatology. Neurol Med Chir (Tokyo).

[22] World Health Organization (1995). Physical status: the use and interpretation of anthropometry. Report of a WHO Expert Committee. WHO Technical Report Series 854.

[23] Rubin DB (1987). Multiple Imputation for Nonresponse in Surveys.

[24] Oichi T, Chikuda H, Ohya J, Ohtomo R, Morita K, Matsui H, Fushimi K, Tanaka S, Yasunaga H. Mortality and morbidity after spinal surgery in patients with Parkinson disease: a retrospective matched-pair cohort study. Spine J. 2016; in press 10.1016/j.spinee.2016.10.024 27884743

[25] Aloisio KM, Swanson SA, Micali N, Field A, Horton NJ (2014). Analysis of partially observed clustered data using generalized estimating equations and multiple imputation. Stata J.

[26] Guijarro R, San Roman CM, Gomez-Huelgas R, Villalobos A, Martin M, Guil M, Martinez-Gonzalez MA, Toledo JB (2010). Impact of dementia on hospitalization. Neuroepidemiology.

[27] Safdar N, Maki DG (2002). The commonality of risk factors for nosocomial colonization and infection with antimicrobial-resistant Staphylococcus aureus, enterococcus, gram-negative bacilli, Clostridium difficile, and Candida. Ann Intern Med.

[28] Fong TG, Tulebaev SR, Inouye SK (2009). Delirium in elderly adults: diagnosis, prevention and treatment. Nat Rev Neurol.

[29] Cohen HJ, Saltz CC, Samsa G, McVey L, Davis D, Feussner JR (1992). Predictors of two-year post-hospitalization mortality among elderly veterans in a study evaluating a geriatric consultation team. J Am Geriatr Soc.

[30] Wieland D, Boland R, Baskins J, Kinosian B (2010). Five-year survival in a Program of All-inclusive Care for Elderly compared with alternative institutional and home- and community-based care. J Gerontol A Biol Sci Med Sci.

[31] Kaye HS, LaPlante MP, Harrington C (2009). Do noninstitutional long-term care services reduce Medicaid spending?. Health Aff.

[32] Golden AG, Roos BA, Silverman MA, Beers MH (2010). Home and community-based Medicaid options for dependent older Floridians. J Am Geriatr Soc.

[33] Zekry D, Herrmann FR, Grandjean R, Vitale AM, De Pinho MF, Michel JP, Gold G, Krause KH (2009). Does dementia predict adverse hospitalization outcomes? A prospective study in aged inpatients. Int J Geriatr Psychiatry.

[34] Menendez ME, Neuhaus V, Bot AG, Vrahas MS, Ring D (2013). Do psychiatric comorbidities influence inpatient death, adverse events, and discharge after lower extremity fractures?. Clin Orthop Relat Res.

[35] Nakanishi M, Shindo Y, Niimura J (2016). Discharge destination of dementia patients who undergo intermediate care at a facility. J Am Med Dir Assoc.

[36] Lin RY, Scanlan BC, Liao W, Nguyen TP (2015). Disproportionate effects of dementia on hospital discharge disposition in common hospitalization categories. J Hosp Med.

[37] Incalzi RA, Gemma A, Marra C, Muzzolon R, Capparella O, Carbonin P (1993). Chronic obstructive pulmonary disease. An original model of cognitive decline. Am Rev Respir Dis.

[38] Pathan SS, Gottesman RF, Mosley TH, Knopman DS, Sharrett AR, Alonso A (2011). Association of lung function with cognitive decline and dementia: the Atherosclerosis Risk in Communities (ARIC) Study. Eur J Neurol.

[39] Davydow DS, Hough CL, Levine DA, Langa KM, Iwashyna TJ (2013). Functional disability, cognitive impairment, and depression after hospitalization for pneumonia. Am J Med.

[40] Shah FA, Pike F, Alvarez K, Angus D, Newman AB, Lopez O, Tate J, Kapur V, Wilsdon A, Krishnan JA, Hansel N, Au D, Avdalovic M, Fan VS, Barr RG, Yende S (2013). Bidirectional relationship between cognitive function and pneumonia. Am J Respir Crit Care Med.

[41] Sellars C, Bowie L, Bagg J, Sweeney MP, Miller H, Tilston J, Langhorne P, Stott DJ (2007). Risk factors for chest infection in acute stroke: A prospective cohort study. Stroke.

